# Integrative Analysis of Fruit Quality and Anthocyanin Accumulation of Plum *cv*. ‘Cuihongli’ (*Prunus salicina* Lindl.) and Its Bud Mutation

**DOI:** 10.3390/plants12061357

**Published:** 2023-03-17

**Authors:** Ling Liao, Yaman Li, Xuejiao Lan, Yiyue Yang, Wen Wei, Jinglan Ai, Xiangning Feng, Hongyu Chen, Yuhang Tang, Lijuan Xi, Zhihui Wang

**Affiliations:** 1 College of Horticulture, Sichuan Agricultural University, Chengdu 611130, China; 13540620948lym@163.com (Y.L.); 18583139221@163.com (X.L.); yyyykl2023@163.com (Y.Y.); ww17844612911@163.com (W.W.); ajl1669737394@163.com (J.A.); 15661305506@163.com (X.F.); 13880498216@163.com (H.C.); 202001613@stu.sicau.edu.cn (Y.T.); 2 Agriculture and Rural Bureau of Qingshen County, Meishan 620000, China; xlj1234562023@163.com

**Keywords:** bud mutation, plum, anthocyanins, enzyme activity, gene expression

## Abstract

Fruit color is one of the quality indicators to judge the freshness of a plum. The coloring process of plum skin is valuable for research due to the high nutritional quality of anthocyanins found in plums. ‘Cuihongli’ (CHL) and its precocious mutant variety ‘Cuihongli Red’ (CHR) were used to analyze the changes of fruit quality and anthocyanin biosynthesis during plum development. The results showed that, during the development of the two plums, the total soluble solid and soluble sugar contents were highest at the mature stage, as the titratable acid trended gradually downward as the fruits of the two cultivars matured, and the CHR fruit showed higher sugar content and lower acid content. In addition, the skin of CHR turned red in color earlier than CHL. Compared with CHL, the skin of CHR had higher anthocyanin concentrations, higher activities of phenylalanine ammonia-lyase (PAL), chalcone isomerase (CHI), dihydroflavonol-4-reductase (DFR), and UDPglucose: flavonoid-3-O-glucosyltransferase (UFGT), and higher transcript levels of genes associated with anthocyanin production. In the flesh of the two cultivars, no anthocyanin content was detected. Taken together, these results suggest that the mutation exerted a major effect on anthocyanin accumulation via modification of the level of transcription; thus, CHR advances the ripening period of ‘Cuihongli’ plum and improves the fruit quality.

## 1. Introduction

Plums (*Prunus salicina* Lindl.) have become one of the most popular stone fruits all over the world due to their strong adaptability, numerous varieties, high economic benefits, and richness in bioactive substances. The major phytochemicals in plums are carotenoids, anthocyanins, flavonols, and vitamin C [1]. There has been evidence that eating fresh or dried plums is good for human health by preventing inflammation [2], hypertension [3], thrombotic risk [4], and cancers [5]. China is the largest plum producer in the world [6], with a planting area >1,900,000 hectares (hm^2^) in 2021 [7]. Most of the cultivated plum cultivars are red-skinned but yellow-fleshed in China [8]. ‘Cuihongli’ is a late-ripening plum variety native to China with red skin and yellow flesh [7].

Color is one of the most distinguishable features among the different fruit species and varieties, and a crucial factor in fruit quality and consumer acceptance [9,10]. There is great variability in both skin and flesh color of plum cultivars [11]. Skin color may be black, purple, red, green, or yellow [12], whilst flesh color can be red or yellow. It is worth mentioning that both colors exist in many shades and some cultivars have a combination of both red and yellow flesh [11]. The research shows that the red pigmentation in plum skin and in the flesh of red-fleshed varieties is mainly caused by anthocyanin accumulation [12,13]. Anthocyanins are the natural protectors for plants; they are widely found in roots, stems, leaves, flowers, fruits, and other organs [14]. With a strong antioxidant capacity, anthocyanins can also prevent the occurrence of coronary diseases and cancer in humans [15]. Anthocyanins are synthesized from a common phenolic precursor through the phenylpropanoid pathway on the cytoplasmic face of the endoplasmic reticulum in plants [8]. In plants, nearly all of the structural genes of the anthocyanin biosynthetic pathway have been cloned [16,17,18], including the expression and regulation of relevant genes [19]. Previous research has shown that the differences in anthocyanin accumulation in fruit were determined primarily by differential levels of anthocyanin gene transcripts [17,20].

Somatic mutations, which are also known as ‘bud sports’, usually occur in woody plant species [21,22]. Up to now, bud sports have been used to select new varieties of grapes [23], pears [16], apples [24], and oranges [25]. These mutants display nearly identical genetic backgrounds as their parents [26,27], and they provide new characteristics while retaining the desirable qualities of the parent plant. The most important economic trait selected from bud sports is the color of the fruit skin, because it is the easiest to recognize [24]. In apple, Li et al. (2018) [22] showed that the accumulation of anthocyanins in the fruit skin causes the bud sport mutants of apple trees with a highly blushed coloring pattern. Zhang et al. (2018) [23] analyzed the changes of anthocyanins biosynthesis of ‘Summer Black’ and its new red flesh mutant during grape berries development, and they found that the occurrence of red flesh might be related to the enhancement of anthocyanins biosynthesis in the whole berry.

In this work, we compared both fruit quality and anthocyanin metabolism between ‘Cuihongli’ (CHL) and its precocious mutant variety ‘Cuihongli Red’ (CHR) at multiple levels, including activities and transcript levels of the key enzymes involved. We chose CHL and CHR plums for this study because CHR is a bud mutation of CHL and the fruit attributes of the two cultivars are essentially the same except for skin color. The results would be helpful for improving agronomic techniques to increase the content of anthocyanins in plums, and revealing the regulatory pathway of skin coloring.

## 2. Results

### 2.1. Changes of the Appearance Index in Plum Cultivars

**Figure 1:**
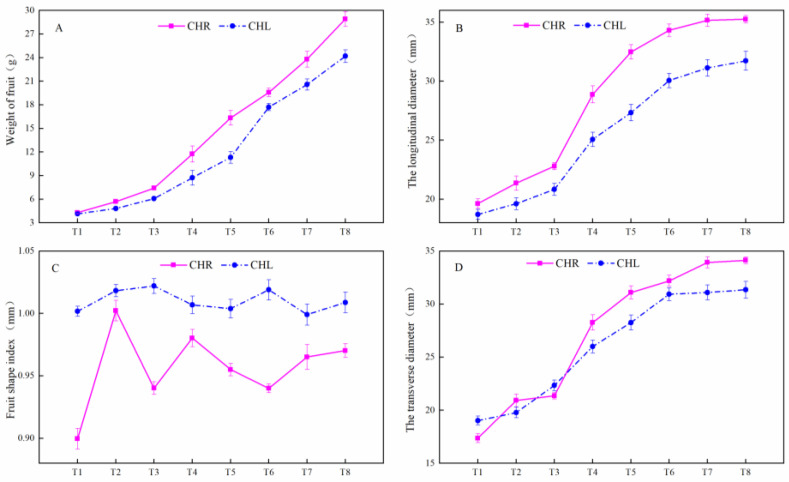
Fruit weights (**A**), longitudinal diameter (**B**), fruit shape (**C**), and transverse diameter (**D**) changes during CHL and CHR plum ripening. Sampling stages T1, T2, T3, T4, T5, T6, T7, and T8 represent 60, 70, 80, 90, 100, 105, 110, and 115 days after flowering, respectively, the same as below. Each value is the mean for three replicates. The vertical bar indicates the standard error.

**Figure 2:**
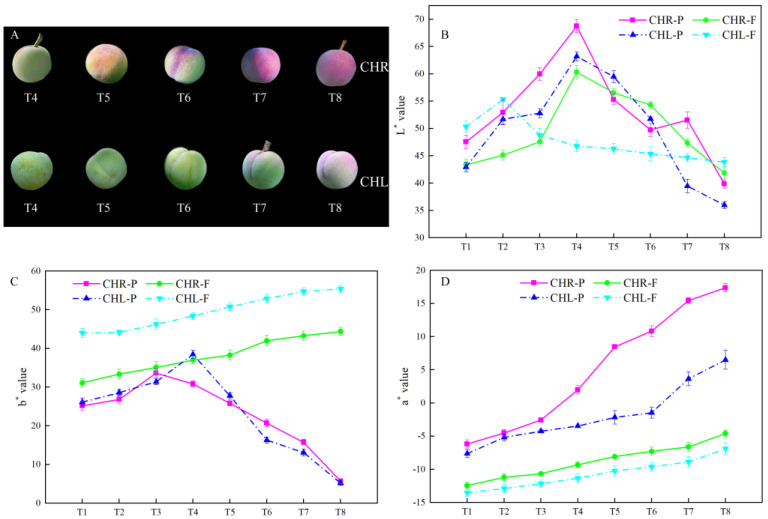
Changes in color during plum development. (**A**) Photographs of T4 to T8 development stages of CHL and CHR; (**B**) Fruit L* value, L* represents brightness; (**C**) Fruit b* value, b* represents yellow–blue difference; (**D**) Fruit a* value, a* represents the red–green difference. CHR-P and CHL-P represent the skin of ‘Cuihongli Red’ and ‘Cuihongli’, respectively. CHR-F and CHL-F represent the flesh of ‘Cuihongli Red’ and ‘Cuihongli’, respectively, the same as below. Each value is the mean for three replicates. The vertical bar indicates the standard error.

With the development of plums, the basic quality indexes of CHL and its mutant (CHR) had similar trends as listed in Figure 1. The single fruit weights, the transverse diameter, and the longitudinal diameter of CHL and CHR increased gradually during growth and development. At maturity, the single fruit weight, transverse diameter, and longitudinal diameter of CHR were significantly greater than those of CHL. The fruit shapes of the two plums were similar, and the fruit shape index was about 0.95–1.1 (Figure 1C). Meanwhile, the plums of the two cultivars gradually became red (Figure 2A). The lightness (L*) values in the fruit skin and flesh were increased first and then decreased, and there was no difference of L* between the two varieties (Figure 2B). The value of a* in both skin and flesh increased as the fruit ripened. It is noteworthy that the a* value of flesh was negative during the whole fruit development stage, while the a* value of CHR skin changed from negative to positive at T4. Furthermore, the a* value of CHL skin changed from negative to positive at T7, whilst the a* value of CHR skin was significantly higher than that of CHL (Figure 2D). The value of b* in skin increased first and then decreased, and there was no difference of b* in the skin between CHR and CHL (Figure 2C). The value of b* in flesh increased as the fruit ripened, and the value of b* in the pulp of CHL was significantly higher than that of CHR.

### 2.2. Changes of the Soluble Sugar Contents, Total Soluble Solids, Total Acid, and Vc Contents in Plum Cultivars

**Figure 3:**
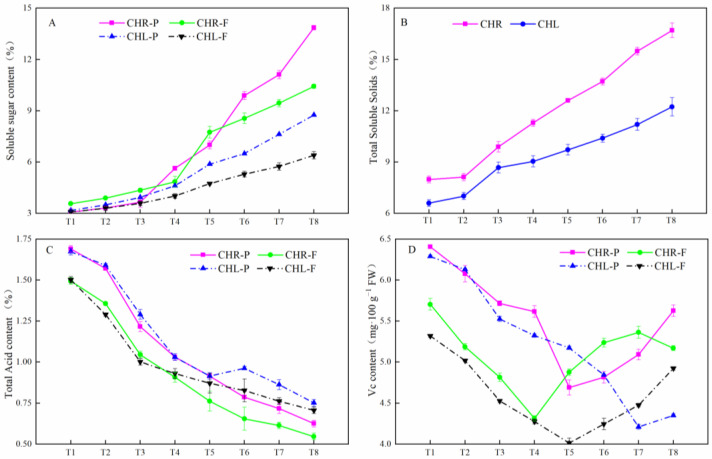
Changes in soluble sugar contents (SS), Total soluble solids (TSS), Total acid contents (TA), and Vc contents during the development of plums. (**A**) SS; (**B**) TSS; (**C**) TA; (**D**) Vc contents. CHR-P and CHL-P represent the skin of ‘Cuihongli Red’ and ‘Cuihongli’, respectively. Each value is the mean for three replicates. The vertical bar indicates the standard error.

The soluble sugar (SS) and total soluble solids (TSS) contents in the pericarp and flesh gradually increased (Figure 3A,B), and the total acid (TA) gradually decreased with the growth and development of the CHL and CHR fruits (Figure 3C). The SS contents in the skin and flesh of CHL were 8.75% and 6.40%, and the TA values were 0.75% and 0.71% at T8, respectively. The SS contents in the skin and flesh of CHR were 13.85% and 10.43%, respectively, which were significantly higher than those in the skin and flesh of CHL, whereas the TA values were 0.63% and 0.55% at T8, respectively, which were significantly lower than the TA content of the peel and pulp of CHL. During fruit development, the TSS in the flesh of CHL was 12.23% at T8, which was significantly lower than that in CHR pulp by 16.73%. The Vc contents in the skin and flesh of the two cultivars (Figure 3D) trended different. At maturation (T8), the Vc content in the skin was higher than that in flesh between the two cultivars, and the highest Vc content was in the CHR skin (5.63 mg·100 g^−1^ FW).

### 2.3. Comparison of Anthocyanin Profiles in the Skin and Flesh of Mature Plums from CHL and CHR

**Figure 4:**
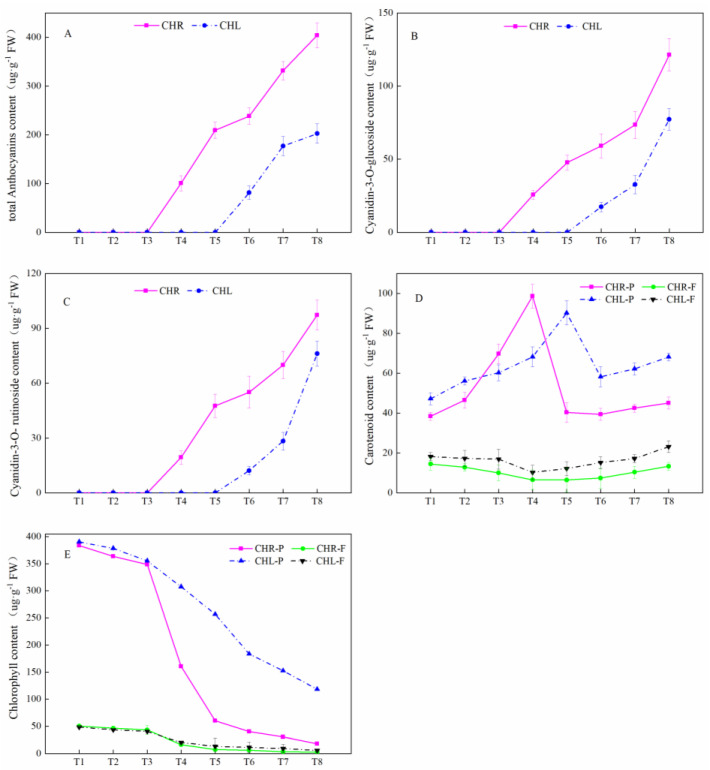
Total anthocyanins content (TMAC), cyanidin−3−O−glucoside (C3G) contents, cyanidin−3−O−rutinoside (C3R) contents, total carotenoid contents, and total chlorophyll content in CHL and CHR during development. (**A**) TMAC; (**B**) C3G; (**C**) C3R; (**D**) Total carotenoid contents and (**E**) Total chlorophyll content. Each value is the mean for three replicates. The vertical bar indicates the standard error.

The change in total anthocyanins content (TMAC) of plum skin is presented in Figure 4A. It should be noted that anthocyanin content was not detected in the flesh of the two plums from CHL and CHR during the whole development period. The TMAC in the skin of CHR began to accumulate from T4, while in CHL, it began to accumulate from T6. The TMAC was significantly changed from T4 to T8 in CHR, from 100.67 μg/g to 403.92 μg/g, and the TMAC in CHR ranged from 81.22 μg/g to 202.68 μg/g from T6 to T8. There are two main components detected in the skins of CHL and CHR during the development (Figure 4B,C), including cyanidin-3-O-glucoside (C3G) and cyanidin-3-O-rutinoside (C3R). Both the C3G and C3R contents of ripe CHR fruits were significantly higher than that of the ripe CHL fruits (*p* < 0.05).

The amounts of total carotenoid in the skin and flesh of CHR and CHL at eight developmental stages were analyzed in Figure 4D. Carotenoid accumulation in the skin of two cultivars during fruit development and ripening was divided into two stages. From T1–T4, the total carotenoid content of the CHR skin increased about 1.6-fold; from T1–T5, the total carotenoid content of the CHL skin increased by 91.1%. After that, the total carotenoid content decreased. The carotenoid content in the flesh was significantly lower than that in the skin, whilst at the fully ripe stage T8, the total carotenoid contents of the flesh of CHR and CHL were 13.32 μg/g and 23.16 μg/g, respectively.

The change trend of chlorophyll content in the skin and flesh showed a downward trend in CHR and CHL (Figure 4E). At the fully ripe stage T8, the chlorophyll contents of the skin of CHR and CHL were 18.29 μg/g and 119.21 μg/g, respectively, about an 18.3-fold difference. The chlorophyll contents of the flesh of CHR and CHL were 2.69 μg/g and 5.63 μg/g, respectively.

### 2.4. Changes of Related Enzyme Activities

**Figure 5:**
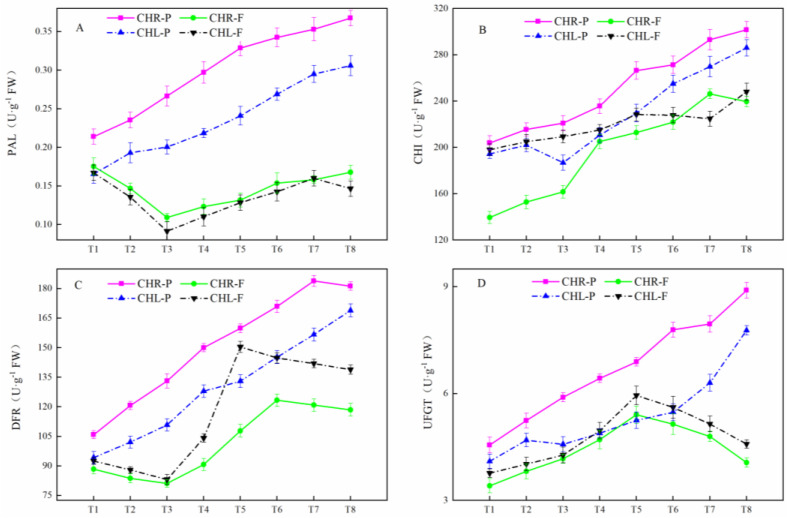
Phenylalanine ammonia−lyase (PAL), chalcone isomerase (CHI), dihydroflavonol−4−reductase (DFR), and UDPglucose: flavonoid−3−O− glucosyltransferase (UFGT) changes during CHL and CHR ripening. (**A**) PAL; (**B**) CHI; (**C**) DFR; (**D**) UFGT. Each value is the mean for three replicates. The vertical bar indicates the standard error.

PAL activity exhibited a different change between the skin and flesh (Figure 5A). The PAL activity in the skin of CHR and CHL tended to increase before and at T8. The change trend of PAL activity in the flesh of CHR and CHL decreased at first and then increased. By comparison, the PAL activity in the skin of CHR was significantly higher than that in CHL at stage T8. The changes in the CHI activity in both the skin and flesh of CHR and CHL exhibited a similar profile, with an increasing trend before and at T8 (Figure 5B). The CHI activity in the skin of CHR was significantly higher at stage T8 than that in CHL. The DFR activity exhibited a very different profile between the skin and flesh (Figure 5C). The DFR activity in CHR skin reached its peak at T7 (183.9 U/g FW), and in CHL skin, reached its peak at T8 (168.9 U/g FW). The DFR activity in the flesh of CHR was maintained at a high level at T6 (123.3 U/g FW), while in the flesh of CHL, it was maintained at a high level at T5 (150.4 U/g FW). UFGT activity in the skin of CHR and CHL showed an increasing trend before and at T8, and reached 8.90 U/g FW and 7.78 U/g FW, respectively (Figure 5D). In the flesh of CHR and CHL, it tended to increase before T5, then decrease quickly until T8 at last (4.06 and 4.58 U/g FW, respectively).

### 2.5. Variations of Gene Expression

**Figure 6:**
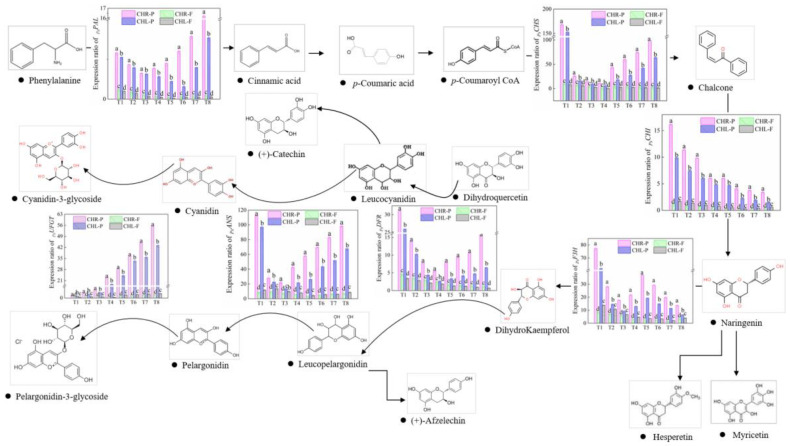
Part of the phenylpropanoid biosynthesis pathway, showing related pathways for flavonoid and anthocyanin synthesis. The general pathway is catalyzed by phenylalanine ammonia-lyase (*_Ps_PAL*), chalcone synthase (*_Ps_CHS*), chalcone isomerise (*_Ps_CHI*), flavanone 3-hydroxylase (*_Ps_F3H*), dihydroflavonol 4-reductase (*_Ps_DFR*), anthocyanidin synthase (*_Ps_ANS*), and UDPglucose:flavonoid−3−O−glucosyltransferase (*_Ps_UFGT*). In addition, the gene expression data are present in the corresponding part. Each value is the mean for three replicates. The vertical bar indicates the standard error. Columns with different letters differ significantly at *p* < 0.05.

In order to investigate the fluctuations in the expression levels of key genes participating in the biosynthesis of anthocyanins during the fruit development of CHL and CHR plums, RT-qPCR was conducted for *_Ps_PAL*, *_Ps_CHS*, *_Ps_CHI*, *_Ps_F3H*, *_Ps_DFR*, *_Ps_ANS*, and *_Ps_UFGT*. Part of the anthocyanin biosynthesis pathway is shown in Figure 6, and this illustrates the variations of the transcript levels of these genes. In general, in the two varieties, similar time–course gene expression patterns during development and ripening were found. Among these genes, *_Ps_PAL*, *_Ps_ANS*, *_Ps_CHS*, and *_Ps_DFR* experienced a transient down-regulation and then up-regulation in the skin and flesh during fruit development or maturation. *_Ps_UFGT* showed increasing expressions whereas the expression of *_Ps_CHI* was sustained or decreased. Notably, the expression of *_Ps_F3H* was down-regulated first, then increased, and then decreased during ripening. The maximum level of expression for *_Ps_PAL* and *_Ps_UFGT* was reached approximately at T8, while the maximum level of expression for the other genes was reached approximately at T1. The skin of CHR showed significantly higher expressions of *_Ps_PAL*, *_Ps_CHS*, *_Ps_CHI*, *_Ps_F3H*, *_Ps_DFR*, *_Ps_ANS*, and *_Ps_UFGT* compared to CHL during development and ripening.

### 2.6. Correlation Analysis

**Figure 7:**
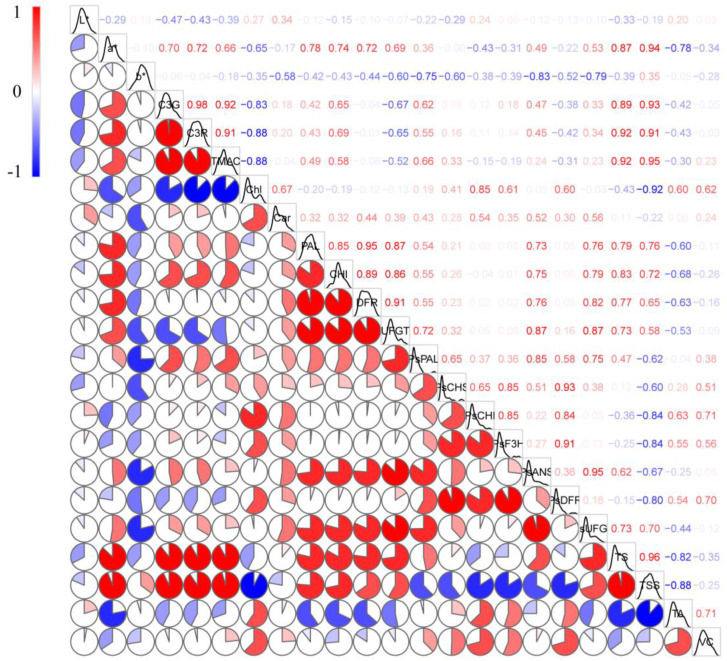
Pearson’s correlation coefficients of fruit quality parameters, pigment content, anthocyanin−synthesis−related enzymes, and gene correlation in CHL and CHR plums. The color bar indicates the correlation coefficient, blue indicates a negative correlation, and red indicates a positive correlation.

The correlation analysis conducted by the Pearson method is shown in Figure 7. Both C3R and C3G exhibited significant positive correlations with a* and no significant negative correlations with L* and b*. In addition, both C3R and C3G exhibited significant positive correlations with total anthocyanins content and significant negative correlations with chlorophyll. Both C3R and C3G exhibited positive correlations with PAL and CHI, while they exhibited negative correlations with UFGT and DFR. The transcript levels of DFR exhibited negative correlations with C3R and C3G. The transcript levels of *_Ps_PAL*, *_Ps_CHS*, *_Ps_CHI*, *_Ps_ANS*, *_Ps_F3H*, and *_Ps_UFGT* showed positive correlations with C3R and C3G, while *_Ps_DFR* showed negative correlations with C3R and C3G. Both TS and TSS showed significant positive correlations with C3R and C3G, while TA and Vc exhibited negative correlations with C3R and C3G.

## 3. Discussion

### 3.1. CHR Demonstrated a High Level of Fruit Quality Compared to CHL

A bud sport can be vegetatively propagated, thereby preserving the novel phenotype without sexual reproduction, and in numerous species, bud sports have been identified to ripen earlier or later than their parents [28]. As listed in Figure 1, the basic quality indexes of ‘Cuihongli’ (CHL) and its mutant (CHR) had similar trends with the ripening of plum fruits. For the fresh market, measurements of TA are important for controlling ripening [29]. Organic acids in CHL and CHR accumulated during earlier stages of development and then decreased at the later stage of ripening (Figure 3), which may be due to the enhanced basic metabolism and synthesis of sugars or secondary compounds in mature fruits [30]. It is noteworthy that the skin of CHR started coloring at the T4 time, while the skin of CHL did not turn red until T7. Previous studies have shown that there is a tight positive correlation between sugar and anthocyanin content [31]; at T4, the skin of CHR showed high levels of TSS and anthocyanins, while the skin of CHL showed a low content of TSS without anthocyanins, which indicated that the mutation mechanism not only promoted the accumulation of carbohydrates, but also increased the anthocyanin accumulation in the skin.

### 3.2. Comparison of Colour in the Skin of CHR and CHL

Color is mainly governed by pigments and its development is the most fascinating and complex phenomenon during ripening [32]. Basically, the color development in the plant system is mainly responsible for anthocyanins, carotenoids, chlorophylls, and betalains [33]. As we all know, anthocyanins can lead to darker fruit colors [34]. However, along with the dominant presence of the anthocyanin pigments, the ripening stages, the high concentrations of flavonols, and the blending of chlorophyll, carotenoids, and anthocyanins also affects the colors [34,35]. In this study, we checked chlorophylls, carotenoids, and anthocyanins in the skin and flesh of CHL and CHR. Comparison of the chlorophyll, carotenoid, and anthocyanin contents in CHR revealed a similar profiling with respect to the CHL (Figure 4); however, the skin of CHR turns red earlier than that of CHL which was the most remarkable phenotypic characteristic of the CHR plum. 

Of various economic traits selected from bud sports, fruit skin color is a key indicator of fruit quality regarding its commercial and nutritional value [28,36]. Ripening (comparison of T1 and T8) of two plum cultivars resulted in significantly increased a* values of the skin (Figure 2). The results also showed a significant influence of bud sports on the a* value. The a* value measured in CHR skin changed from negative to positive at T4, while in CHL skin, it changed from negative to positive at T7, consistent with the accumulation of anthocyanin content in the skin. The redness of fruit skin is determined by the types and contents of anthocyanins [36]. Our results demonstrated that the content of total anthocyanins in the CHR skin was higher than that in the CHL skin, and with the development of the plum, the anthocyanin content showed upward trends. The color of the CHR fruits was, at the T4 picking time point, more developed compared with CHL, which proved that CHR fruits at T4 were more mature than CHL.

The chlorophyll absorption might be a certain method to determine the real maturation stage of the fruits [37]. When we compared the chlorophyll content of the two plum varieties studied, a decreasing trend was found in the skin and flesh, as shown in Figure 4. Statistically significant differences (*p* < 0.05) were observed as a function of the harvesting phase after T4, which is similar with the findings of Alina et al. (2018) [38], which corresponds physiologically and horticulturally with the growth and maturation of the fruit.

### 3.3. Comparison of Anthocyanin Profiles in the Skin of Mature Plums from CHR and CHL

The biosynthesis of anthocyanins is achieved via the phenylpropanoid pathway metabolism in plants [39]. Anthocyanin concentration was found to increase during plum ripening, which imparted dark hues to the fruit skin [40]. Statistically significant differences were ascertained among cultivars in our study. The highest content of total anthocyanins was at T8 measured in CHR. Similar patterns were also found in the total anthocyanin content and PAL, CHI, DFR, and UFGT activity in CHL and CHR plums sampled at different stages, indicating that skin reddening is closely related to the enhancement of the phenylpropanoid pathway metabolism, in which PAL is the key initial gateway enzyme catalyzing the biosynthesis of anthocyanins and non-anthocyanin pheolic compounds. Cyanidin-3-O-glucoside and -rutinoside are some of the major polyphenols in plums with red skin and/or flesh [41]. In our study, the major anthocyanin was cyanidin-3-O-glucoside. 

Considering that cyanidin-3-O-glucoside confers a red hue to fruits [42], it might be the crucial phytochemical leading to skin reddening [43]. The expression profile of anthocyanin biosynthetic genes in the plum skin has proved this hypothesis. Our research found that the increased expression levels of most unigenes, such as *PsPAL*, *PsCHS*, *PsCHI*, *PsF3H*, *PsDFR*, *PsANS*, and *PsUFGT*, observed during the colored stage, showed a positive correlation with anthocyanin content, which was consistent with previous research by Hyun et al. (2014) [44]. The upstream genes in the anthocyanin biosynthetic pathway exhibited an obvious increase in expression in the skin of CHR compared with CHL, indicating that the mutation exerted a major effect on anthocyanin accumulation via modification of the level of transcription.

## 4. Materials and Methods

### 4.1. Plant Material

Four-year-old trees of ‘Cuihongli’ plum (CHL) and its bud mutation, ‘Cuihongli Red’ plum (CHR), grafted on *P. persica* rootstock were used in this study. Trees were planted at a spacing of 3 m × 4 m in Danling County, Sichuan Province (30°04′ N, 103°53′ W). The annual average temperature was 16~18 °C and the annual rainfall was 1200~1500 mm. Each cultivar had three three-tree replicates distributed in a randomized complete block design. Trees were selected for uniform fruit density. During the experimentation, all trees received the same amounts of nitrogen, phosphorus, and potassium fertilizers. The plant protection in the orchards followed standard horticultural disease and pest control.

Fruit samples of CHL and CHR were collected from eight developmental stages of 60, 70, 80, 90, 100, 105, 110, and 115 days after flowering, and these sampling stages were called T1, T2, T3, T4, T5, T6, T7, and T8, respectively. Thirty fruits from each cultivar were used for the determination of fruit characteristics at each stage. Fruits were transported to the laboratory on the day of harvest and then were divided into two groups. One group used eighteen samples for physiological index assessment. After the measurement of TSS, single fruit weight, vertical diameter, and transverse diameter index, the peel and flesh were isolated from same batch of fruit and were used for the determination of chlorophyll, carotenoid, and total anthocyanins contents. From the second group, the peel and flesh were isolated, and the flesh was frozen immediately in liquid nitrogen and stored at −80 °C for subsequent analyses [i.e., high-performance liquid chromatography (HPLC) analysis, enzyme activity, and quantitative real-time (qRT)−PCR].

### 4.2. Determination of Fruit Characteristics

Single fruit weight was measured with an electronic balance, whilst the length and diameter of the fruit were measured with vernier calipers. Fruit shape index represents the ratio of fruit length to diameter. The color indices L*, a*, and b* (L* represents brightness, a* represents the red–green difference, and b* represents yellow–blue difference) of each fruit were measured at the equatorial position of the skin and flesh using a colorimeter (CR-400). Total soluble solids (TSS) were measured with a hand-held refractometer (Atago Co., Ltd., Japan) with the unit of %. Titration with a sodium hydroxide indicator was used to determine the titratable acid (TA) content of the fruits. The anthrone–sulfuric-acid method was used to determine the soluble sugar content (%). The Vc was measured using 2.6-dichlorophenol-indophenol titration.

### 4.3. Anthocyanin Analysis

The total anthocyanin content was quantified by the pH differential method as described by Diaconeasa et al. (2015) [45]. Briefly, the total absorbance of the extracts was measured at 520 and 700 nm in pH 1.0 and 4.5 buffers; the final absorbance of anthocyanins was estimated using the formula: TAc = *A* × MW × 5 × 100 × *V*/ε, where TAc stands for total anthocyanin content (in milligrams per 100 g, ascyanidin-3-O-glucose equivalent), *V* for final volume (in milliliters), and *A* = (A_520nm_ − A_700nm_ pH 1.0) − (A_520nm_ − A_700nm_ pH 4.5). A molar absorptivity (ε) of 26,900 and a molecular weight (MW) of 449.2 were used according to Wrolstand et al. (1982) [46]. Three technical replicates were used.

Anthocyanins were analyzed as in Fang et al. (2021) [8] with HPLC. Briefly, anthocyanins were extracted from 2 g freeze-dried ground powder, in the presence of methanol containing 0.1 % HCl (*v*/*v*). HPLC analysis was performed using an Agilent 1260 system (Agilent Technologies, Sacramento, CA, USA). Separation was performed using a C_18_ column (4.6 mm × 250 mm, 5 µm, CNW Technologies, Shanghai, China). The mobile phases were eluent A (0.3% phosphoric acid in water) and eluent B (acetonitrile) at a flow rate of 1 mL min^−1^. The linear gradient of phase B was as follows: 0–20 min, 5%; 20–30 min, 15%; 30–40 min, 25%; 40–40 min, 25%; 48–50 min, 5%. The injection volume was 10 µL. Anthocyanin components were detected at 520 nm and peaks were identified by comparison of peaks to authentic standards.

### 4.4. Spectrophotometric Determination of Chlorophyll and Carotenoid Content

A mixture of 80% acetone and anhydrous ethanol (1:1) was prepared for chlorophyll and carotenoid extraction. Samples of 0.5 g were placed in test tubes containing 10 mL of the prepared solution, and were left in the dark for 24 h. Immediately afterwards, the absorptions were measured by a spectrophotometer (Meipuda Instrument Co., Ltd., China) at a wavelength of 480 nm (carotenoids), 649 nm (chlorophyll b), and 665 nm (chlorophyll a). Concentrations of chlorophylls a and b and carotenoids in the extract were determined by using Wellburn (1994) [47] equations.

### 4.5. Determination of Enzyme Activity

Phenylalanine ammonia-lyase (PAL) activity was extracted from flesh tissue and assayed according to the method of Li et al. (2013) [48]. The PAL activity was defined as the amount of protein causing an increase of 0.01 in absorbance at 290 nm per min.

Chalcone isomerase (CHI) activity was assayed according to Lister et al. (1996) [49], with minor modifications. Flesh tissue (1 g) was homogenized with 0.1 g PVP and 5.0 mL of 50 mM phosphate buffer (pH 7.0, containing 50 mM sodium ascorbate, 18 mM *β*-mercaptoethanol). The homogenate was centrifuged at 12,000× *g* for 20 min at 4 °C. The CHI activity was defined as the amount of protein causing a decrease of 0.01 in absorbance at 381 nm per min.

The dihydroflavonol 4-reductase (DFR) activity was assayed according to the method of Duan et al. (2019) [50]. Flesh tissue (1.0 g) was homogenized at 4 °C with 5 mL of 100 mM Tris-HCl buffer (pH 7.5, containing 5 mM dithiothreotol). The homogenate was centrifuged as described above and the supernatant was used for the enzyme assay. The DFR activity was defined as the amount of protein causing an increase of 0.01 in absorbance at 550 nm per min.

Extraction and assay of the activities of UFGT was according to the report by Lister et al. (1996) [49], with minor modifications. Flesh tissue (1.0 g) was homogenized with 5.0 mL acetone precooled at −20 °C. The homogenate was centrifuged at 10,000× *g* for 20 min at 4 °C and the residue was homogenized again with 5.0 mL of 100 Mm boric acid buffer (pH 8.8, containing 5 mM sodium ascorbate). The homogenate was centrifuged as described above and the supernatant was used as crude enzyme. The UFGT activity was defined as the amount of protein causing an increase of 0.01 in absorbance at 351 nm per min.

### 4.6. RNA Extraction and Gene Expression Analysis

RNA extraction and qRT−PCR were performed as previously described (Zhang et al., 2011). There were three biological replicates of samples from each period in our experiment. The actin gene was used as an internal reference in the real-time PCR experiments to normalize the mRNA levels in each sample. The relative expression levels of the target genes were calculated using the 2^−ΔΔCt^ formula. All primer sequences are listed in Table 1.

### 4.7. Data Analysis

All data were analyzed by using SPSS 21.0 (SPSS Inc., Chicago, IL, USA). One-way ANOVA and Duncan’s multiple comparison test were used to analyze the differences between samples at different stages of development and ripening.

## 5. Conclusions

‘Cuihongli Red’ (CHR) is a bud-independent mutation derived from ‘Cuihongli’ (CHL). Its characteristic is that the pericarp turns red in advance. Differences in anthocyanin concentrations in the skin of CHR and CHL are closely related to their PAL, CHI, DFR, and UFGT activities. Higher expression levels of *PsPAL*, *PsCHS*, *PsCHI*, *PsF3H*, *PsDFR*, *PsANS*, and *PsUFGT* and higher activities of PAL, CHI, DFR, and UFGT are responsible for the higher concentrations of anthocyanins in the skin of CHR. Compared with CHL, the bud mutation not only enhanced anthocyanin synthesis in the skin of CHR, but also increased TSS and total sugar, decreased total acid, and changed the ripening period of CHR fruit, making the fruit mature ahead of time. The fruits of CHR and CHL show similar fruit shapes and appearances. The mutant variety CHR provides new characteristics while retaining the desirable qualities of the CHL, and the fruit quality of the CHR fruits was substantially better than in the CHL cultivar.

## Figures and Tables

**Table 1:** Target genes analyzed by RT-qPCR.

Gene Name	Primer Sequence (5′–3′)
*Actin*	Forward: GCAGACAGGATGAGCAAGGAGATTAC
	Reverse: TCTGTTGGAAGGTACTGAGGGATG
*PsPAL*	Forward: CCTCCCACAGAAGAACAAAGCAAG
	Reverse: GCCTGACTCTTTCGTGCTCCC
*PsCHS*	Forward: GCGGACTACCAGCTCACCAAG
	Reverse: CACACAACAAGAACACGAGCAC
*PsCHI*	Forward: AGGTGACAACGATACTGCCA
	Reverse: CGCCAGGTGGGAAGTTTT
*PsF_3_H*	Forward: CAATGGGAGGTTCAAGAATG
	Reverse: TCTGGAATGTGGCTATGG
*PsDFR*	Forward: GGCTGACCTGGCGGACGAG
	Reverse: CACTTCGTTCTCGGGGTCTTTGG
*PsANS*	Forward: GAGTACATCAGACCCAAGGAAGAGC
	Reverse: GCCTTCTTCAACTCCTCCCTGC
*PsUFGT*	Forward: GTACCCTTCTTATGGTCTCTCA
	Reverse: CCATCCCATTGCTTGCTTT

## Data Availability

All data generated or analyzed during this study are included in this published article.
